# London Water Supply

**Published:** 1899-01-28

**Authors:** 


### London Water Supply.

The issue last week of the first interim report of the Commission on the London Water Supply serves to emphasise one of the most important factors in the problem, namely, the inadequacy of the Thames to provide more water than is already abstracted from it unless a very much larger provision for storage be made than at present exists. The natural storage in the deeper strata cannot be relied on for much assistance, for as was insisted on by Lord Farrar last Saturday, at a meeting of the Surrey County Council, all the counties around London are feeling it wrong that the water companies should sink deep wells in their land and pump them dry for the benefit of Londoners and for the benefit of the dividends of the water companies. Nevertheless, the report of the Commission, while making clear the necessity of providing intercommunication between the mains of the different companies, with the object of preventing the occurrence of local "water famines," has also made it clear that such rearrangement of the pipes goes but a little way to remedy the difficulty, for, as matters stand, all the companies together have not got any considerable excess of water to distribute. One company has a little more than another, but if the full supply were to be kept up through the summer the margin would be but small, and, if we read between the lines of the report, we see that London has far more nearly reached the end of its tether in regard to water than many people imagine. Storage then is essential, and now the attempt is being made to narrow the question down to this point alone, and to maintain that the whole problem is merely one of where (*i.e.*, in Wales or on the banks of the Thames) can we most cheaply store ?

This view was very cleverly stated last month by the Special Correspondent of the *Times*, who, as some would say, put the whole matter into a nutshell. "The present supply," said he, "is either bad or good. If it is bad, it should be given up entirely, which is not proposed; if it is good enough to keep at all, it is good enough altogether. And as a matter of fact it is proved to be excellent by every possible test." It was also pointed out that the partial abandonment of the Welsh scheme and the evidence already existing that the water of the Thames Valley might be stored as easily as that of the distant mountain streams, reduced the whole question (as between the hybrid scheme and the plan of keeping entirely to the rivers) to one of expense; and we were told that the difference in the cost of the two schemes was what we were to pay for "sentiment and soft water."

We are bound then to inquire what is the "sentiment" which is thus laughed at. The sentiment is one in favour of getting one's water supply from as pure a source as possible, and, looking at the matter from a public health point of view, we say advisedly that so long as science does not enable us to tell with accuracy when water is pure, we cannot afford to neglect the old dictum of a former head of the public health service, that if we want to make sure of the safety of water supplies we "must go . . . in search of the conditions surrounding water sources."

Now, we need hardly say that the conditions of the Thames valley, highly cultivated and manured as it is, and dotted over with towns of considerable size, while the river which drains it is itself a public water-way bearing on its surface a large floating population, would utterly condemn it as a source of supply were we not tolerably confident of being able to manufacture from the drainage of this valley a water fit to drink. Fortunately we have every reason to believe—nay, we may say we have proof—that by careful filtration it is possible to purify even such water as the Thames and Lea provide. Unfortunately we have equal reason to believe that by the means at present in use this purification is not continuously effected, and we are afraid it will have to be confessed that no one knows with any approach to exactitude what it would cost to produce a constantly good effluent from the filter beds. Under these circumstances, however well satisfied some people may affect to be with sources the pollution of which is likely to go on increasing with increasing population, we do not think that the statement that if the Thames "is good enough to keep at all it is good enough altogether" expresses the whole truth. A side of the problem which ought never to be lost sight of is the question of quality, and when estimates of expense are being considered the greatest care should be taken to ensure that equal qualities of water are being compared. Looking at the matter then from a health point of view it is not merely a question of expense as between the two plans—the Thames and the Welsh rivers—but of the expense of getting an equally pure water from each source, and in regard to that the present inquiry has produced curiously little evidence.

